# 12-(4-Chloro­phen­yl)-7-methyl-10-phenyl-3,4,5,6,8,10-hexa­aza­tricyclo­[7.3.0.0^2,6^]dodeca-1(9),2,4,7,11-penta­ene

**DOI:** 10.1107/S1600536810048373

**Published:** 2010-11-27

**Authors:** Rina D. Shah, Mukesh M. Jotani, Edward R. T. Tiekink

**Affiliations:** a Department of Chemistry, M.G. Science Institute, Navrangpura, Ahmedabad, Gujarat 380 009, India; b Department of Physics, Bhavan’s Sheth R.A. College of Science, Ahmedabad, Gujarat 380 001, India; c Department of Chemistry, University of Malaya, 50603 Kuala Lumpur, Malaysia

## Abstract

The 12 non-H atoms defining the triple-fused-ring system in the title compound, C_19_H_13_ClN_6_, are almost coplanar (r.m.s. deviation = 0.023 Å). The chloro-substituted ring is almost effectively coplanar with the central atoms [dihedral angle = 6.74 (13)°], but the N-bound benzene ring is not [dihedral angle = 54.38 (13)°]. In the crystal, supra­molecular chains along the *a* axis sustained by C—H⋯π and π–π [centroid–centroid distance between N_4_C and C_4_N five-membered rings = 3.484 (2) Å] stacking occur. A very long C—Cl⋯π contact is also seen.

## Related literature

For biological activity of imidazoles, see: Yohjiro *et al.* (1990 ▶). For related structures, see: Jotani *et al.* (2010*a* ▶,*b* ▶). Semi-empirical quantum chemical calculations were performed using *MOPAC2009*, see: Stewart (2009 ▶).
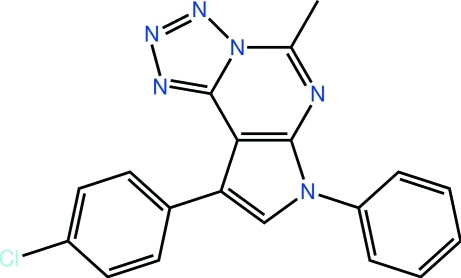


## Experimental

### 

#### Crystal data

C_19_H_13_ClN_6_
*M* *_r_* = 360.80
Orthorhombic, 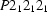

*a* = 6.9459 (5) Å
*b* = 9.7010 (8) Å
*c* = 24.0382 (16) Å
*V* = 1619.7 (2) Å^3^
*Z* = 4
Mo *K*α radiation
μ = 0.25 mm^−1^
*T* = 293 K
0.40 × 0.22 × 0.15 mm

#### Data collection

Bruker SMART APEX CCD diffractometer
Absorption correction: multi-scan (*SADABS*; Sheldrick, 1996 ▶) *T*_min_ = 0.928, *T*_max_ = 0.975
8751 measured reflections
1677 independent reflections
1405 reflections with *I* > 2σ(*I*)
*R*_int_ = 0.047

#### Refinement

*R*[*F* ^2^ > 2σ(*F* ^2^)] = 0.035
*wR*(*F* ^2^) = 0.096
*S* = 0.98
1677 reflections
236 parameters
H-atom parameters constrained
Δρ_max_ = 0.17 e Å^−3^
Δρ_min_ = −0.22 e Å^−3^

### 

Data collection: *APEX2* (Bruker, 2004 ▶); cell refinement: *APEX2* and *SAINT* (Bruker, 2004 ▶); data reduction: *SAINT* and *XPREP* (Bruker, 2004 ▶); program(s) used to solve structure: *SHELXS97* (Sheldrick, 2008 ▶); program(s) used to refine structure: *SHELXL97* (Sheldrick, 2008 ▶); molecular graphics: *ORTEP-3* (Farrugia, 1997 ▶) and *DIAMOND* (Brandenburg, 2006 ▶); software used to prepare material for publication: *publCIF* (Westrip, 2010 ▶).

## Supplementary Material

Crystal structure: contains datablocks global, I. DOI: 10.1107/S1600536810048373/hb5751sup1.cif

Structure factors: contains datablocks I. DOI: 10.1107/S1600536810048373/hb5751Isup2.hkl

Additional supplementary materials:  crystallographic information; 3D view; checkCIF report

## Figures and Tables

**Table 1:** Hydrogen-bond geometry (Å, °) *Cg*1 and *Cg*2 are the centroids of the C14–C19 and C8–C13 rings, respectively.

*D*—H⋯*A*	*D*—H	H⋯*A*	*D*⋯*A*	*D*—H⋯*A*
C7—H7a⋯*Cg*1^i^	0.96	2.62	3.509 (5)	154
C17—Cl1⋯*Cg*2^ii^	1.74 (1)	3.61 (1)	4.423 (4)	106 (1)

Symmetry codes: (i) 
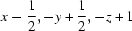
; (ii) 
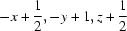
.

**Fig. 1.**
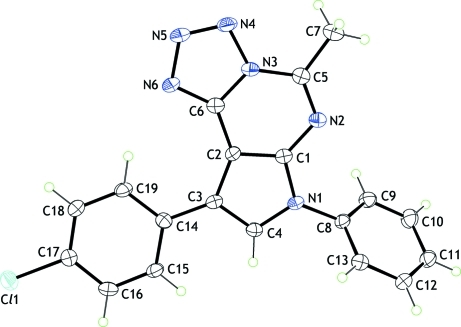
The molecular structure of (I) showing displacement ellipsoids at the 35% probability level.

**Fig. 2.**
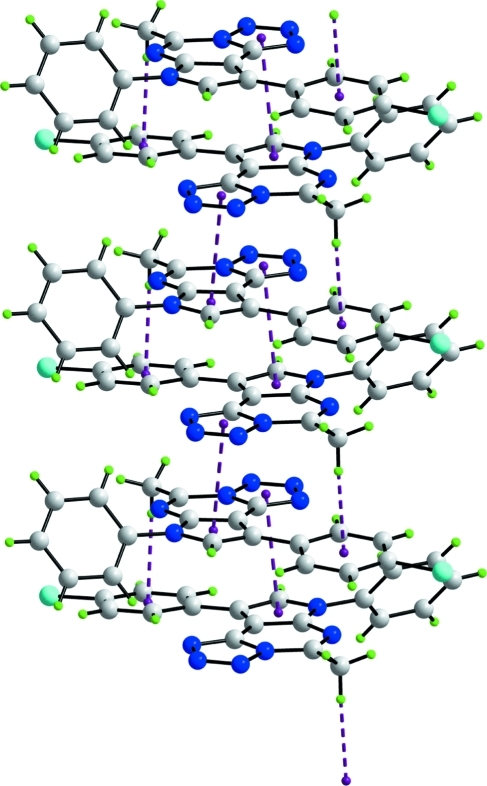
A supramolecular chain aligned along the a axis in (I), mediated by C–H···π and π–π interactions, both shown as purple dashed lines.

## supplementary crystallographic information

### Comment

The crystal structure of the title compound, (I), was examined in connection with on-going structural studies of imidazoles (Jotani *et al.*, 2010*a*; Jotani *et al.*, 2010*b*), which are known to possess a wide spectrum of biological activities such as herbicidal, anti-bacterial, anti-fungal, *etc*. (Yohjiro *et al.*, 1990).

In (I), the 12 non-hydrogen atoms comprising the three ring fused system are co-planar with a r.m.s. deviation of 0.023 Å [max. and min. deviations = 0.033 (3) Å for atom N1 and -0.039 (4) Å for C3]. Whereas the chloro-substituted benzene ring is co-planar with the fused ring system [the C2–C3–C14–C15 torsion angle = -173.1 (4) °], the N-bound benzene ring is twisted out of the plane [the C1–N1–C8–C9 torsion angle = -54.0 (6) °]. Other features in the molecule match recently determined literature precedents (Jotani *et al.*, 2010*a*; Jotani *et al.*, 2010*b*)

The presence of C—H···π, Table 1, and π–π interactions between five-membered rings [ring centroid(N1,C1–C4)···ring centroid(N3–N6,C6) = 3.484 (2) Å with an angle of inclination = 2.2 (2) ° for *i*: 1/2 + *x*, 1/2 - *y*, 1 - *z*] lead to supramolecular chains along the *a* axis. The major interactions involving the Cl atom are of the type C—Cl···π, Table 1, which serve to connect molecules along the *b* axis.

Semi-empirical Quantum Chemical Calculations were performed using the MOPAC2009 programme (Stewart, 2009) to optimize the experimental structure with the Parametrization Model 6 (PM6) approximation together with restricted the Hartree Folk closed shell wavefunction; the minimizations were terminated at a r.m.s. gradient less than 0.01 kJ-mol^-1^ Å^-1^. These calculations gave an optimized structure which had different conformations for the chloro-substituted and the N-bound benzene rings, as seen in the C2—C3—C14—C15 and C1—N1—C8—C9 torsion angles of 146.1 and -38.5 °, respectively.

### Experimental

To a well stirred mixture of 2-methyl-4-chloro-5-(4-chlorophenyl)-7-phenyl-7*H*-pyrrolo[2,3-*d*]pyrimidine (5 mmol) and Aliquat 336 (0.202 g, 0.5 mmol) in toluene (25 ml) was added sodium azide (0.390 g, 6 mmol) in water (5 ml). The reaction mixture was stirred under reflux conditions for 1–1.5 h. Thereafter, the two phases were separated, the aqueous phase was extracted with toluene (15 ml) and combined organic layers were washed with water (10 *x* 2 ml) and passed through anhydrous sodium sulfate. The excess of solvent was distilled under reduced pressure. The oily residue was treated with cold methanol. The obtained solid was filtered, dried, and crystallized from dioxane to yield colourless blocks; m.pt: 251–253 K.

### Refinement

The C-bound H atoms were geometrically placed (C–H = 0.93–0.96 Å) and refined as riding with *U_iso_*(H) = 1.2–1.5*U_eq_*(parent atom). In the absence of significant anomalous scattering effects, 1165 Friedel pairs were averaged in the final refinement. In the final refinement a low angle reflection evidently effected by the beam stop was omitted, *i.e*. (002).

### Crystal data

**Table:**

C_19_H_13_ClN_6_	*F*(000) = 744
*M**_r_* = 360.80	*D*_x_ = 1.480 Mg m^−^^3^
Orthorhombic, *P*2_1_2_1_2_1_	Mo *K*α radiation, λ = 0.71073 Å
Hall symbol: P 2ac 2ab	Cell parameters from 3699 reflections
*a* = 6.9459 (5) Å	θ = 2.3–29.6°
*b* = 9.7010 (8) Å	µ = 0.25 mm^−^^1^
*c* = 24.0382 (16) Å	*T* = 293 K
*V* = 1619.7 (2) Å^3^	Block, colourless
*Z* = 4	0.40 × 0.22 × 0.15 mm

### Data collection

**Table:**

Bruker SMART APEX CCD diffractometer	1677 independent reflections
Radiation source: fine-focus sealed tube	1405 reflections with *I* > 2σ(*I*)
graphite	*R*_int_ = 0.047
ω and φ scans	θ_max_ = 25.0°, θ_min_ = 1.7°
Absorption correction: multi-scan (*SADABS*; Sheldrick, 1996)	*h* = −8→4
*T*_min_ = 0.928, *T*_max_ = 0.975	*k* = −11→11
8751 measured reflections	*l* = −27→28

### Refinement

**Table:**

Refinement on *F*^2^	Primary atom site location: structure-invariant direct methods
Least-squares matrix: full	Secondary atom site location: difference Fourier map
*R*[*F*^2^ > 2σ(*F*^2^)] = 0.035	Hydrogen site location: inferred from neighbouring sites
*wR*(*F*^2^) = 0.096	H-atom parameters constrained
*S* = 0.98	*w* = 1/[σ^2^(*F*_o_^2^) + (0.066*P*)^2^] where *P* = (*F*_o_^2^ + 2*F*_c_^2^)/3
1677 reflections	(Δ/σ)_max_ < 0.001
236 parameters	Δρ_max_ = 0.17 e Å^−^^3^
0 restraints	Δρ_min_ = −0.22 e Å^−^^3^

### Special details

**Table:**

Geometry. All s.u.'s (except the s.u. in the dihedral angle between two l.s. planes) are estimated using the full covariance matrix. The cell s.u.'s are taken into account individually in the estimation of s.u.'s in distances, angles and torsion angles; correlations between s.u.'s in cell parameters are only used when they are defined by crystal symmetry. An approximate (isotropic) treatment of cell s.u.'s is used for estimating s.u.'s involving l.s. planes.
Refinement. Refinement of *F*^2^ against ALL reflections. The weighted *R*-factor *wR* and goodness of fit *S* are based on *F*^2^, conventional *R*-factors *R* are based on *F*, with *F* set to zero for negative *F*^2^. The threshold expression of *F*^2^ > 2σ(*F*^2^) is used only for calculating *R*-factors(gt) *etc*. and is not relevant to the choice of reflections for refinement. *R*-factors based on *F*^2^ are statistically about twice as large as those based on *F*, and *R*- factors based on ALL data will be even larger.

### Fractional atomic coordinates and isotropic or equivalent isotropic displacement parameters (Å^2^)

**Table:**

	*x*	*y*	*z*	*U*_iso_*/*U*_eq_	
Cl1	0.39113 (13)	0.63434 (9)	0.75124 (3)	0.0425 (3)	
N1	0.4515 (4)	0.4484 (3)	0.42147 (10)	0.0298 (6)	
N2	0.4434 (4)	0.2102 (3)	0.39545 (10)	0.0298 (6)	
N3	0.4374 (4)	0.0610 (3)	0.47095 (10)	0.0283 (6)	
N4	0.4365 (5)	−0.0648 (3)	0.49607 (11)	0.0376 (7)	
N5	0.4374 (5)	−0.0389 (3)	0.54866 (12)	0.0425 (8)	
N6	0.4393 (5)	0.0980 (3)	0.56050 (11)	0.0361 (7)	
C1	0.4475 (5)	0.3117 (3)	0.43459 (12)	0.0278 (7)	
C2	0.4411 (5)	0.2987 (3)	0.49267 (11)	0.0245 (7)	
C3	0.4383 (5)	0.4353 (3)	0.51533 (12)	0.0273 (7)	
C4	0.4465 (5)	0.5218 (3)	0.47005 (13)	0.0301 (7)	
H4	0.4484	0.6175	0.4722	0.036*	
C5	0.4366 (5)	0.0842 (3)	0.41388 (13)	0.0300 (7)	
C6	0.4393 (5)	0.1602 (3)	0.51112 (12)	0.0281 (7)	
C7	0.4230 (6)	−0.0361 (4)	0.37716 (14)	0.0405 (9)	
H7A	0.2920	−0.0674	0.3758	0.061*	
H7B	0.5037	−0.1085	0.3912	0.061*	
H7C	0.4645	−0.0111	0.3404	0.061*	
C8	0.4640 (5)	0.5089 (3)	0.36712 (12)	0.0289 (8)	
C9	0.6114 (5)	0.4716 (4)	0.33131 (13)	0.0349 (8)	
H9	0.7011	0.4051	0.3416	0.042*	
C10	0.6221 (6)	0.5346 (4)	0.28045 (13)	0.0408 (9)	
H10	0.7186	0.5087	0.2557	0.049*	
C11	0.4940 (5)	0.6351 (4)	0.26511 (13)	0.0394 (9)	
H11	0.5054	0.6785	0.2308	0.047*	
C12	0.3477 (5)	0.6713 (4)	0.30125 (13)	0.0396 (9)	
H12	0.2596	0.7390	0.2912	0.048*	
C13	0.3322 (5)	0.6070 (4)	0.35236 (13)	0.0358 (8)	
H13	0.2329	0.6304	0.3765	0.043*	
C14	0.4292 (5)	0.4834 (3)	0.57356 (12)	0.0276 (7)	
C15	0.4074 (5)	0.6233 (3)	0.58553 (13)	0.0315 (7)	
H15	0.3997	0.6860	0.5564	0.038*	
C16	0.3968 (5)	0.6708 (3)	0.63951 (13)	0.0336 (8)	
H16	0.3824	0.7644	0.6467	0.040*	
C17	0.4079 (5)	0.5776 (3)	0.68284 (12)	0.0300 (7)	
C18	0.4282 (5)	0.4389 (3)	0.67246 (13)	0.0340 (8)	
H18	0.4342	0.3768	0.7019	0.041*	
C19	0.4397 (5)	0.3921 (3)	0.61828 (12)	0.0319 (7)	
H19	0.4548	0.2983	0.6115	0.038*	

### Atomic displacement parameters (Å^2^)

**Table:**

	*U*^11^	*U*^22^	*U*^33^	*U*^12^	*U*^13^	*U*^23^
Cl1	0.0529 (6)	0.0401 (5)	0.0344 (4)	−0.0031 (4)	0.0028 (4)	−0.0066 (4)
N1	0.0367 (16)	0.0210 (15)	0.0317 (14)	−0.0009 (13)	0.0002 (12)	0.0045 (12)
N2	0.0289 (15)	0.0257 (16)	0.0348 (14)	−0.0007 (14)	−0.0003 (12)	−0.0003 (12)
N3	0.0281 (15)	0.0209 (14)	0.0358 (14)	−0.0017 (12)	0.0016 (12)	−0.0010 (12)
N4	0.0465 (19)	0.0210 (15)	0.0453 (18)	−0.0009 (15)	0.0005 (14)	0.0049 (13)
N5	0.061 (2)	0.0213 (17)	0.0457 (18)	−0.0007 (16)	0.0016 (16)	0.0061 (14)
N6	0.0501 (18)	0.0232 (16)	0.0349 (16)	−0.0033 (14)	0.0033 (13)	0.0065 (12)
C1	0.0252 (17)	0.0243 (18)	0.0339 (17)	0.0003 (15)	0.0006 (14)	0.0015 (14)
C2	0.0231 (17)	0.0235 (17)	0.0269 (15)	0.0006 (15)	0.0011 (13)	0.0036 (13)
C3	0.0260 (17)	0.0229 (17)	0.0329 (17)	0.0016 (15)	−0.0016 (14)	0.0033 (14)
C4	0.0365 (19)	0.0214 (18)	0.0323 (17)	−0.0015 (15)	0.0007 (15)	−0.0013 (14)
C5	0.0238 (18)	0.0308 (19)	0.0355 (18)	0.0000 (15)	0.0017 (14)	−0.0022 (15)
C6	0.0233 (17)	0.0260 (18)	0.0350 (17)	0.0001 (15)	0.0020 (13)	0.0004 (14)
C7	0.043 (2)	0.030 (2)	0.048 (2)	0.0006 (18)	0.0033 (18)	−0.0072 (16)
C8	0.0356 (19)	0.0227 (18)	0.0286 (17)	−0.0026 (15)	−0.0030 (14)	0.0036 (14)
C9	0.035 (2)	0.031 (2)	0.0384 (18)	0.0043 (16)	0.0018 (15)	0.0023 (16)
C10	0.044 (2)	0.045 (2)	0.0336 (19)	−0.0009 (19)	0.0070 (15)	0.0038 (17)
C11	0.054 (2)	0.031 (2)	0.0338 (19)	−0.0061 (17)	−0.0049 (15)	0.0061 (17)
C12	0.053 (2)	0.032 (2)	0.0336 (18)	0.0090 (17)	−0.0063 (16)	0.0038 (16)
C13	0.043 (2)	0.031 (2)	0.0330 (18)	0.0033 (16)	0.0013 (14)	−0.0008 (16)
C14	0.0256 (18)	0.0259 (18)	0.0314 (16)	−0.0047 (15)	0.0011 (14)	−0.0022 (14)
C15	0.038 (2)	0.0224 (17)	0.0341 (17)	0.0005 (16)	−0.0012 (14)	0.0057 (15)
C16	0.035 (2)	0.0216 (18)	0.0443 (19)	−0.0005 (14)	0.0002 (15)	−0.0034 (16)
C17	0.0268 (18)	0.033 (2)	0.0304 (16)	−0.0015 (14)	−0.0010 (13)	−0.0010 (14)
C18	0.041 (2)	0.0290 (19)	0.0325 (17)	−0.0014 (17)	0.0019 (15)	0.0067 (15)
C19	0.0369 (18)	0.0220 (18)	0.0366 (18)	−0.0036 (16)	−0.0021 (15)	0.0037 (14)

### Geometric parameters (Å, °)

**Table:**

Cl1—C17	1.738 (3)	C8—C13	1.368 (5)
N1—C1	1.364 (4)	C8—C9	1.386 (5)
N1—C4	1.368 (4)	C9—C10	1.369 (5)
N1—C8	1.435 (4)	C9—H9	0.9300
N2—C5	1.301 (4)	C10—C11	1.370 (5)
N2—C1	1.362 (4)	C10—H10	0.9300
N3—C6	1.364 (4)	C11—C12	1.382 (5)
N3—N4	1.361 (4)	C11—H11	0.9300
N3—C5	1.390 (4)	C12—C13	1.382 (5)
N4—N5	1.289 (4)	C12—H12	0.9300
N5—N6	1.359 (4)	C13—H13	0.9300
N6—C6	1.332 (4)	C14—C19	1.395 (4)
C1—C2	1.402 (4)	C14—C15	1.395 (4)
C2—C6	1.415 (4)	C15—C16	1.379 (4)
C2—C3	1.433 (4)	C15—H15	0.9300
C3—C4	1.376 (4)	C16—C17	1.382 (4)
C3—C14	1.477 (4)	C16—H16	0.9300
C4—H4	0.9300	C17—C18	1.375 (5)
C5—C7	1.466 (4)	C18—C19	1.382 (4)
C7—H7A	0.9600	C18—H18	0.9300
C7—H7B	0.9600	C19—H19	0.9300
C7—H7C	0.9600		
			
C1—N1—C4	108.0 (3)	C13—C8—N1	118.7 (3)
C1—N1—C8	127.6 (3)	C9—C8—N1	120.2 (3)
C4—N1—C8	124.5 (3)	C10—C9—C8	118.6 (3)
C5—N2—C1	116.4 (3)	C10—C9—H9	120.7
C6—N3—N4	108.6 (2)	C8—C9—H9	120.7
C6—N3—C5	125.8 (3)	C9—C10—C11	121.5 (3)
N4—N3—C5	125.7 (3)	C9—C10—H10	119.2
N5—N4—N3	105.1 (3)	C11—C10—H10	119.2
N4—N5—N6	113.3 (3)	C10—C11—C12	119.3 (3)
C6—N6—N5	104.9 (3)	C10—C11—H11	120.4
N2—C1—N1	122.9 (3)	C12—C11—H11	120.4
N2—C1—C2	128.5 (3)	C11—C12—C13	120.1 (3)
N1—C1—C2	108.5 (3)	C11—C12—H12	120.0
C1—C2—C6	113.4 (3)	C13—C12—H12	120.0
C1—C2—C3	107.2 (3)	C8—C13—C12	119.5 (3)
C6—C2—C3	139.4 (3)	C8—C13—H13	120.2
C4—C3—C2	105.2 (3)	C12—C13—H13	120.2
C4—C3—C14	124.0 (3)	C19—C14—C15	117.7 (3)
C2—C3—C14	130.8 (3)	C19—C14—C3	121.9 (3)
N1—C4—C3	111.0 (3)	C15—C14—C3	120.5 (3)
N1—C4—H4	124.5	C16—C15—C14	121.7 (3)
C3—C4—H4	124.5	C16—C15—H15	119.2
N2—C5—N3	119.2 (3)	C14—C15—H15	119.2
N2—C5—C7	123.0 (3)	C17—C16—C15	119.2 (3)
N3—C5—C7	117.7 (3)	C17—C16—H16	120.4
N6—C6—N3	108.1 (3)	C15—C16—H16	120.4
N6—C6—C2	135.2 (3)	C18—C17—C16	120.6 (3)
N3—C6—C2	116.7 (3)	C18—C17—Cl1	119.2 (2)
C5—C7—H7A	109.5	C16—C17—Cl1	120.1 (3)
C5—C7—H7B	109.5	C17—C18—C19	119.9 (3)
H7A—C7—H7B	109.5	C17—C18—H18	120.0
C5—C7—H7C	109.5	C19—C18—H18	120.0
H7A—C7—H7C	109.5	C18—C19—C14	121.0 (3)
H7B—C7—H7C	109.5	C18—C19—H19	119.5
C13—C8—C9	121.0 (3)	C14—C19—H19	119.5
			
C6—N3—N4—N5	0.2 (4)	N4—N3—C6—C2	179.8 (3)
C5—N3—N4—N5	−179.9 (3)	C5—N3—C6—C2	−0.1 (5)
N3—N4—N5—N6	−0.1 (4)	C1—C2—C6—N6	177.8 (4)
N4—N5—N6—C6	0.1 (4)	C3—C2—C6—N6	−2.2 (8)
C5—N2—C1—N1	−178.9 (3)	C1—C2—C6—N3	−2.2 (4)
C5—N2—C1—C2	−1.5 (5)	C3—C2—C6—N3	177.9 (4)
C4—N1—C1—N2	177.4 (3)	C1—N1—C8—C13	128.3 (4)
C8—N1—C1—N2	−4.1 (5)	C4—N1—C8—C13	−53.5 (5)
C4—N1—C1—C2	−0.4 (4)	C1—N1—C8—C9	−54.1 (5)
C8—N1—C1—C2	178.1 (3)	C4—N1—C8—C9	124.2 (4)
N2—C1—C2—C6	3.2 (5)	C13—C8—C9—C10	−0.4 (5)
N1—C1—C2—C6	−179.1 (3)	N1—C8—C9—C10	−178.0 (3)
N2—C1—C2—C3	−176.8 (3)	C8—C9—C10—C11	1.6 (5)
N1—C1—C2—C3	0.9 (4)	C9—C10—C11—C12	−1.6 (6)
C1—C2—C3—C4	−1.0 (4)	C10—C11—C12—C13	0.4 (5)
C6—C2—C3—C4	179.0 (4)	C9—C8—C13—C12	−0.8 (5)
C1—C2—C3—C14	179.1 (3)	N1—C8—C13—C12	176.8 (3)
C6—C2—C3—C14	−1.0 (7)	C11—C12—C13—C8	0.8 (5)
C1—N1—C4—C3	−0.3 (4)	C4—C3—C14—C19	−173.7 (3)
C8—N1—C4—C3	−178.8 (3)	C2—C3—C14—C19	6.2 (6)
C2—C3—C4—N1	0.8 (4)	C4—C3—C14—C15	7.0 (5)
C14—C3—C4—N1	−179.3 (3)	C2—C3—C14—C15	−173.1 (3)
C1—N2—C5—N3	−1.1 (5)	C19—C14—C15—C16	0.1 (5)
C1—N2—C5—C7	177.3 (3)	C3—C14—C15—C16	179.5 (3)
C6—N3—C5—N2	2.0 (5)	C14—C15—C16—C17	−0.1 (5)
N4—N3—C5—N2	−178.0 (3)	C15—C16—C17—C18	−0.3 (5)
C6—N3—C5—C7	−176.5 (3)	C15—C16—C17—Cl1	−179.0 (3)
N4—N3—C5—C7	3.5 (5)	C16—C17—C18—C19	0.7 (5)
N5—N6—C6—N3	0.0 (4)	Cl1—C17—C18—C19	179.3 (3)
N5—N6—C6—C2	−179.9 (4)	C17—C18—C19—C14	−0.6 (5)
N4—N3—C6—N6	−0.1 (4)	C15—C14—C19—C18	0.2 (5)
C5—N3—C6—N6	179.9 (3)	C3—C14—C19—C18	−179.1 (3)

### Hydrogen-bond geometry (Å, °)

**Table:**

Cg1 and Cg2 are the centroids of the C14–C19 and C8–C13 rings, respectively.

**Table:**

*D*—H···*A*	*D*—H	H···*A*	*D*···*A*	*D*—H···*A*
C7—H7a···Cg1^i^	0.96	2.62	3.509 (5)	154
C17—Cl1···Cg2^ii^	1.737 (4)	3.608 (2)	4.423 (4)	106.34 (13)

Symmetry codes: (i) *x*−1/2, −*y*+1/2, −*z*+1; (ii) −*x*+1/2, −*y*+1, *z*+1/2.
